# Frailty and hospital outcomes among patients with neurological disorders

**DOI:** 10.1007/s10072-025-08144-4

**Published:** 2025-04-03

**Authors:** Marco Toccaceli Blasi, Fabrizio Raffaele, Daniele Belvisi, Simona Buscarnera, Giuseppe Bruno, Giovanni Fabbrini, Marco Canevelli

**Affiliations:** 1 Department of Human Neuroscience, “Sapienza” University, Viale dell’Università 30, Rome, 00185 Italy; 2 Barcelonaβeta Brain Research Center (BBRC), Pasqual Maragall Foundation, Barcelona, Spain; 3 IRCCS Neuromed, Pozzilli, IS Italy; 4 Santa Lucia Foundation IRCCS, Rome, Italy; 5 National Center for Disease Prevention and Health Promotion, Italian National Institute of Health, Rome, Italy; 6 Aging Research Center, Department of Neurobiology, Care Sciences and Society, Karolinska Institutet and Stockholm University, Stockholm, Sweden

**Keywords:** Frailty, Neurological disease, Emergency department assessment, Prognosis

## Abstract

**Introduction:**

Patients with neurological disorders, particularly those who are chronologically and biologically older, may display highly varied clinical courses and trajectories. The present study explored the association between frailty and hospital outcomes among patients with acute neurological presentations admitted to an Italian university hospital.

**Materials and methods:**

A cross-sectional study considered all patients consecutively admitted to the Neurology Unit of the Policlinico Umberto I University Hospital of Rome (Italy). A 40-item Frailty Index (FI) was retrospectively developed based on the clinical information collected in the Emergency Department (ED). Data on hospitalization outcomes were prospectively collected during the patient’s stay at the Neurology Unit. Linear and logistic regression models were conducted to test the association between FI and hospital outcomes.

**Results:**

Overall, 185 participants (women 50.3%; mean age 68.6, SD 18.6 years) were included. FI scores ranged between 0 and 0.43, with a median value of 0.15 [IQR 0.10], and were positively correlated with age (Spearman’s rho 0.55, *p* < 0.001). In a linear regression model adjusted by age, sex, and diagnosis, FI was significantly associated with the number of days spent in the Neurology Unit (B 2.18, 95%CI 0.25–4.11, per 0.1 increase; *p* = 0.03). In bivariate logistic regression models adjusted by age, sex, and diagnosis, increasing FI scores were significantly associated with a lower likelihood of being discharged at home (OR 0.37, 95%CI 0.20–0.63, per 0.1 increase; *p* < 0.001), with higher odds of nosocomial infections (OR 1.67, 95%CI 1.05–2.73 per 0.1 increase; *p* = 0.03), and prescription of antibiotics (OR 1.77, 95%CI 1.11–2.92, per 0.1 increase; *p* = 0.02).

**Conclusion:**

Frailty is adversely associated with hospital outcomes in patients with acute neurological disorders. Assessing frailty could improve patient stratification, prognostication, and care planning, with a relevant impact on healthcare resources.

## Introduction

Neurological disorders rank among the leading causes of morbidity, disability, and mortality worldwide [1]. The complexity of these conditions arises from their varying phenotypic presentation, the multisystemic involvement, the multifaceted underlying pathophysiological mechanisms, and the potential for diverse outcomes. This clinical intricacy is further exacerbated by population aging [2] and the increasing average age of patients presenting with neurological illnesses [3]. As people age, they tend to accumulate multiple chronic diseases and deficits, resulting in conditions of multimorbidity and frailty [4]. The intricate interplay between neurological diseases and such age-related conditions of homeostatic disruption, somatic burden, and poor health can significantly modify clinical course and health outcomes, making care planning even more challenging [5]. Therefore, innovative models and practices are urgently needed to understand and address the evolving needs of chronologically and biologically older patients with neurological disorders.

Frailty is an age-related condition of reduced homeostatic and biological reserves, leading to increased vulnerability to stressors and a higher risk of negative health outcomes [6]. Frailty, traditionally linked to cognitive decline and dementia, is gaining recognition in the field of neurology as a valuable framework for understanding variations in individuals’ susceptibility to negative outcomes [7]. Indeed, there is mounting evidence that it may influence the risk, clinical expression, and outcomes of common neurological disorders [8–14]. Among different frailty operationalizations, the deficit accumulation model postulates that the individual’s degree of frailty is proportional to the number of health deficits accumulated during the life course [15]. For each person, a frailty index (FI) can be calculated as the ratio of accrued deficits to total deficits, thus providing a continuous measure of frailty levels [16]. A FI can be easily computed from a non-fixed set of clinical variables and can be retrospectively created from existing datasets [17]. Such flexible and versatile characteristics have contributed to its growing adoption in clinical practice [18]. While this instrument was initially developed in geriatrics to quantify the degree of frailty in older people, its use now extends to patients with neurological illnesses [7–14].

The emergency department (ED) is typically the first clinical contact for a patient with acute neurological problems. Evaluating frailty in the ED through time-saving and easy-to-use tools [19] can offer valuable insights into a patient’s premorbid condition and risk profile, particularly for those presenting with neurological issues. This assessment can significantly impact care optimization, inform decisions regarding treatment intensity, and enhance shared decision-making [20].

The present study explored the association between frailty and hospital outcomes among patients with acute neurological presentations admitted to an Italian university hospital.

## Materials and methods

This study was performed and reported following STROBE (Strengthening the Reporting of OBservational Studies in Epidemiology) recommendations for cross-sectional studies.

### Setting and subjects

A cross-sectional study was conducted by considering all patients consecutively admitted to the Neurology Unit of the Policlinico Umberto I University Hospital of Rome (Italy) between June 15, 2023, and October 16, 2023. All patients who (i) had accessed the hospital’s ED for an acute neurological disturbance and (ii) had subsequently been transferred to the Neurology Unit were enrolled in the present study. Patients were instead excluded if they met either of the following criteria: (i) they had been transferred to the Neurology Unit from other departments within the hospital (i.e., not directly from the ED), except for the neurovascular treatment unit, or (ii) they were discharged to another hospital or a different ward within the same hospital at the end of their stay in the Neurology Unit. These exclusion criteria were established to ensure that we could accurately measure the baseline frailty status of patients at their first contact with the hospital and to gather detailed information about their outcomes during their hospital stay.

### Frailty

For each participant, a 40-item Frailty Index (FI) was retrospectively constructed by following a standard procedure [21] based on the clinical information collected in the ED. Specifically, a panel of neurologists selected 40 health deficits (i.e., comorbidities, signs, symptoms, and laboratory abnormalities) routinely recorded in the ED assessment and documented for all patients subsequently discharged to other hospital units (Table 1). The validated cutoff of 0.25 [22] was used to divide frail (i.e., FI≥0.25) and non-frail (i.e., FI < 0.25) patients.

### Hospitalization outcomes

Data on hospitalization outcomes were prospectively collected during the patient’s stay at the Neurology Unit through interviews with healthcare providers and a review of medical records. The following outcomes were explored: (i) death; (ii) nosocomial infections, defined as infections occurring 48 h or more after admission to the hospital, which did not appear to be incubating at the time of admission [23]; (iii) delirium, assessed using the Confusion Assessment Method [24]; (iv) prescription of antibiotics; (v) prescription of sedatives (i.e., any drug, such as benzodiazepines and antipsychotics, purposely used to induce sedation); (vi) use of physical restraints; (vii) falls. Furthermore, data on length of stay (in days) and discharge destination (i.e., home, rehabilitation, long-term care, and hospice) were collected at the end of the hospital stay in the Neurology Unit.

### Covariates

All models evaluated age, sex, and neurological diagnosis as potential confounders and were included as covariates. Age was measured in years. Sex was considered as a self-reported binary variable (i.e., woman/man). Neurological diagnoses made at discharge from the Neurology Unit were evaluated and grouped into homogeneous clinical syndromes.

### Statistical analysis

Descriptive statistics were calculated for the total sample and reported as absolute numbers and percentages or medians and interquartile ranges [IQR]. Differences in FI scores by sex were ascertained using the Mann-Whitney U test. Chi-square tests were used to compare the frequency of clinical outcomes between frail and non-frail patients. Differences in length of stay by frailty categories were ascertained using the Mann-Whitney U test. Spearman’s correlation coefficients were used to test the direction and strength of the correlation between FI, age, and hospital length of stay. A linear regression model adjusted by age, sex, and neurological diagnosis (i.e., dummy variable) was conducted to test the association between the FI (continuous) and hospital length of stay. Multiple logistic regression models, adjusted by age, sex, and neurological diagnosis, were performed to test the association between the FI and the explored hospital outcomes (bivariate dependent variables of interest). These latter models were also adjusted for the length of hospital stay. In the regression models, FI scores were multiplied by 10 to facilitate the interpretation of the results indicating the change in the associations for each increase of 0.1 in FI scores, equivalent to an additional four deficits. Statistical significance was set at *p* < 0.05. Statistical analyses were conducted using RStudio v.2024.04.2 (Build 764) per macOS (Posit Software, PBC, 2024).

## Results

A total of 212 participants were considered. However, 27 individuals were excluded based on the established criteria. Specifically, 15 patients had been transferred to the Neurology Unit from other departments rather than directly from the ED, and 12 patients were discharged to other hospital wards at the end of their stay in our unit. As a result, 185 participants (women 50.3%; mean age 68.6, SD 18.6 years) were finally included in the analysis. FI scores ranged between 0 and 0.43, with a median value of 0.15 [IQR 0.10], and positively correlated with age (Spearman’s rho 0.55, *p* < 0.001). Based on the established FI cutoff of 0.25 [22], 31 participants (16.8%) were identified as frail (i.e., FI ≥ 0.25) and 154 (83.2%) as non-frail (i.e., FI < 0.25). The most common neurological diagnoses at discharge were ischemic stroke (38.4%), epilepsy (17.8%), and traumatic brain injury (10.3%). No significant differences in FI scores were observed based on sex. Most patients (75.1%) were discharged at home, and the median length of hospital stay was 8.0 [IQR 6.5] days. Further details regarding the characteristics and hospitalization outcomes of the study population are provided in Table 2.

**Fig. 1:**
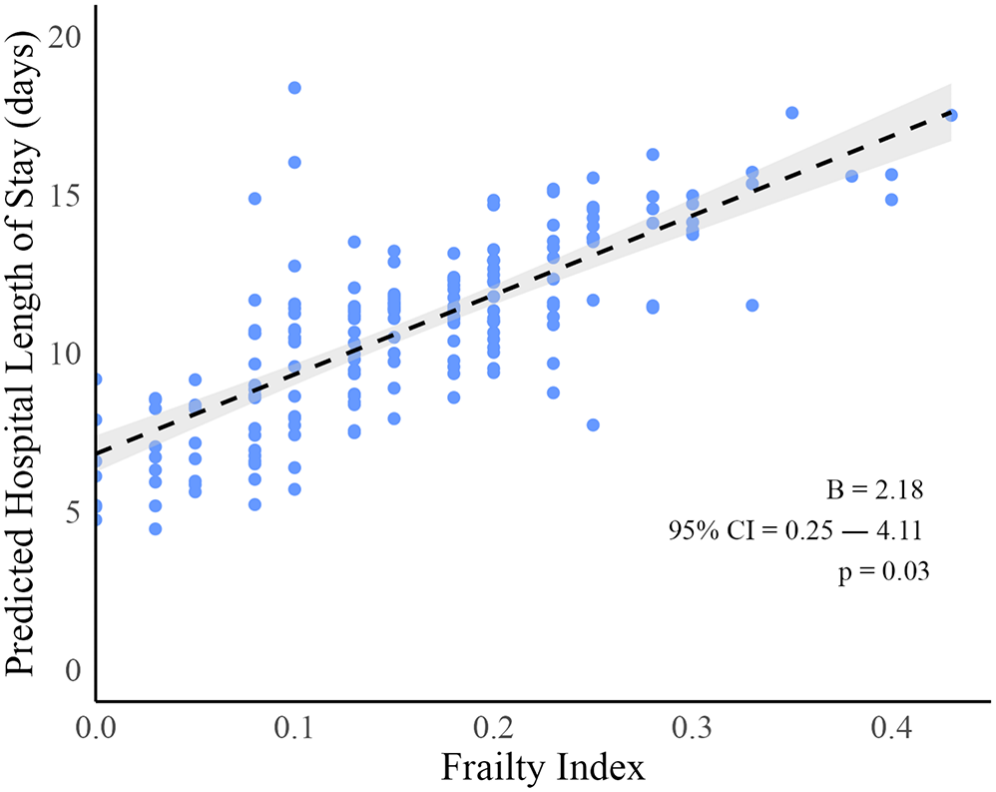
Predicted hospital length of stay (days) by the 40-item Frailty Index in a linear regression model adjusted by age, sex, and neurological diagnosis

**Fig. 2:**
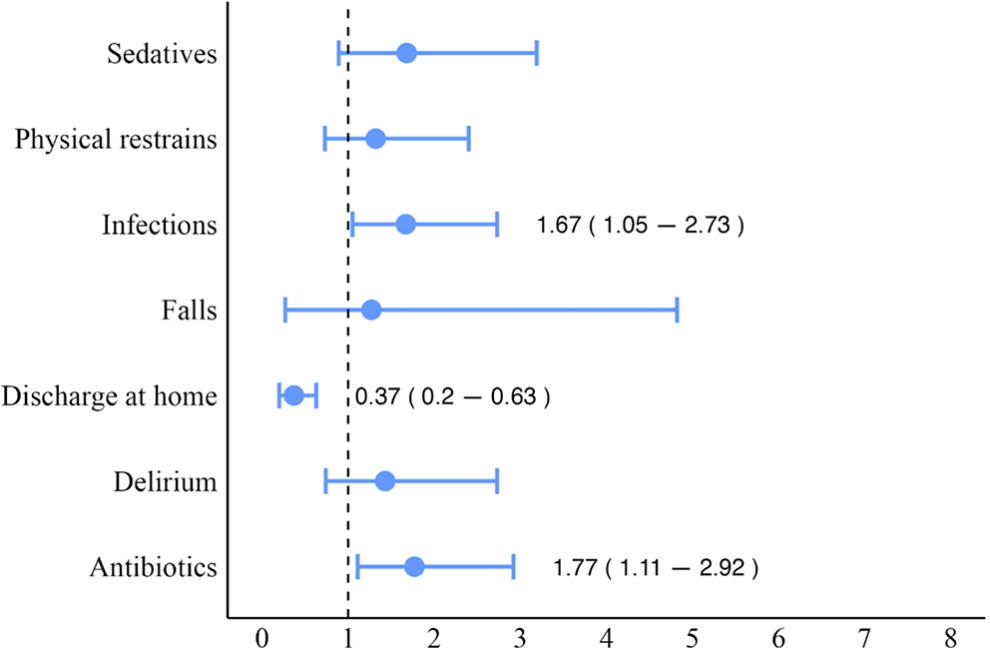
Forest plot of logistic regression models adjusted by age, sex, and neurological diagnosis exploring the impact of the 40-item Frailty Index (independent variable on interests) on multiple hospitalization outcomes (bivariate dependent variables). Data are shown as odds ratios and 95% confidence intervals per 0.1 increase in the Frailty Index

Frailty (i.e., FI ≥ 0.25) was significantly associated with several adverse hospitalization outcomes. Patients classified as frail exhibited a higher frequency of nosocomial infections (χ² = 4.885, *p* = 0.03), antibiotic administration (χ² = 5.713, *p* = 0.02), sedative use (χ² = 7.697, *p* < 0.01), and delirium (χ² = 4.732, *p* = 0.03) compared to non-frail. Additionally, frail patients exhibited longer hospital length of stay (12.0 [9.0] vs. 8.0 [6.0], W = 1561.5, *p* < 0.01) and were less frequently discharged home (χ² = 6.958, *p* < 0.01). FI scores positively correlated with hospital length of stay (Spearman’s rho 0.35, *p* < 0.001). In a linear regression, FI was significantly associated with the number of days spent in the neurological ward (B 2.18, 95%CI 0.25–4.11, per 0.1 increase; *p* = 0.03) (Fig. 1). In bivariate logistic regression, increasing FI scores were significantly associated with a lower likelihood of being discharged at home (OR 0.37, 95%CI 0.20–0.63, per 0.1 increase; *p* < 0.001), with higher odds of nosocomial infections (OR 1.67, 95%CI 1.05–2.73 per 0.1 increase; *p* = 0.03), and prescription of antibiotics (OR 1.77, 95%CI 1.11–2.92, per 0.1 increase; *p* = 0.02). Moreover, a trend toward statistical significance was found for the association between FI and in-hospital mortality (OR 6.27, 95%CI 1.12–91.46, per 0.1 increase; *p* = 0.06). Conversely, no significant association was found between baseline FI and falls, prescription of sedatives, physical restraints, and delirium (all p values > 0.05)(Fig. 2).

After adjusting the logistic regression models for length of stay, the previously identified negative association between FI scores and the odds of being discharged home was confirmed (OR 0.46, 95% CI 0.24–0.83; *p* < 0.01). However, the associations between frailty and the other outcomes discussed earlier did not reach statistical significance (all p-values > 0.05).

## Discussion

The present cross-sectional study aimed to investigate how frailty is associated with hospital outcomes in patients with acute neurological illnesses. Overall, we found that increasing frailty degrees are associated with poorer outcomes, including a longer in-hospital stay, a lower likelihood of being discharged at home, and a higher risk of nosocomial infections.

Our findings align with the existing evidence indicating frailty as a consistent predictor of longer hospital stays and poorer outcomes among hospitalized older people, regardless of chronological age and the underlying disease or condition responsible for hospital admission [25, 26]. Measures that capture a person’s overall clinical and biological complexity, such as the FI, could aid in predicting health trajectories during acute events. A recent systematic review and meta-analysis of nearly 39 million hospital admissions found moderate to severe frailty associated with a 2-3-fold increased risk of hospital stays lasting more than eight days and discharge to locations other than home [26]. Another systematic review found that frailty at hospital admission increases the risk of in-hospital mortality, prolonged hospital stays, functional decline at discharge, and medium- to long-term mortality [25]. Accordingly, there is growing recognition that frailty assessment in emergency care settings can serve as a triage tool to orient clinical decisions, implement tailored healthcare pathways, and properly allocate available resources [19].

To our knowledge, our study is the first to demonstrate the detrimental role of frailty among in-patients with acute neurological conditions admitted to a general neurological ward. Previous studies have already documented the association between frailty and adverse health outcomes (e.g., mortality, functional decline, poorer quality of life) among patients with specific neurological disorders, such as stroke and traumatic brain injury [10, 27]. The current broader emphasis on overall neurological illnesses holds significant clinical and public health importance, as these conditions are among the leading causes of ED visits and hospitalizations in older adults [28]. Therefore, identifying and implementing new tools for stratifying risk profiles and adapting care pathways for these patients assumes special relevance. The possibility of providing prognostic information from the first contact with the ED also has significant repercussions in terms of logistics and operational efficiency. For example, our study suggests that it may be possible to predict since the beginning of the patient journey which departments/wards might have a lower patient turnover and require a greater allocation of resources based on the degrees of frailty (i.e., clinical complexity) of the admitted patients. Having timely information on the frailty status of admitted patients can also facilitate the prompt activation of adapted care pathways, possibly codeveloped with geriatricians, in the premise of a neuro-geriatric approach [7]. In this regard, evidence suggests that targeted interventions, such as comprehensive geriatric assessment, early mobilization, structured physical rehabilitation programs, and nutritional optimization, can help improve outcomes in frail hospitalized patients. Multidisciplinary approaches incorporating these strategies have been shown to reduce hospital-acquired complications, improve functional recovery, and potentially decrease the length of stay [29, 30].

It is important to note that, in our study, frailty assessment was solely based on information typically collected in the emergency setting, without any modifications to standard clinical practice. This approach aligns with the recently proposed principles for frailty screening in the ED, which should be based on a multidimensional evaluation, capturing the patient’s pre-morbid frailty status (i.e., from approximately 2 to 4 weeks before the current illness), and effectively identifying patients at high risk of adverse outcomes [19]. Frailty screens should also be quick to administer [19]. In this regard, other tools may be even more suitable for providing a time-saving frailty assessment in patients with acute neurological presentations. For instance, the Clinical Frailty Scale (CFS) is a widely used alternative to the FI in hospital settings, allowing clinicians to quickly categorize patients into one of nine baseline health states (i.e., from very fit to terminally ill) to orient clinical decisions [31]. This alternative assessment method offers a more pragmatic approach with fewer data collection requirements.

Several limitations of our study should be acknowledged and discussed. First, the cross-sectional design does not allow for exploration of the temporal and causal relationships between frailty and the outcomes studied. While our findings suggest an association between frailty and adverse outcomes, this relationship may be confounded by prolonged hospitalization. Distinguishing between early- and late-onset outcomes could have helped disentangle the relative contributions of frailty and length of stay. However, due to data limitations, we were unable to perform this analysis. Future prospective longitudinal studies could provide precious insights to clarify these associations by capturing changes over time and establishing potential causal links. Second, the relatively small sample size may have limited the statistical power necessary to detect potential associations between frailty and various health outcomes, such as mortality, delirium, and falls, already documented by previous research [32–35]. Moreover, the limited subgroup size did not allow us to perform stratified analysis. Third, since FI items were retrospectively collected from ED records, some frailty-related deficits may have been underreported or misclassified. We ultimately could not include measures of socioeconomic status or social vulnerability in our analysis. These factors, along with frailty, likely contributed to certain hospitalization outcomes, such as prolonged length of stay [36–38]. Not including them in the analysis may have limited our ability to identify their impact on the development of these outcomes. In conclusion, frailty is adversely associated with hospital outcomes in patients with acute neurological disorders. Assessing frailty could improve patient stratification, prognostication, and care planning for these patients. Future longitudinal studies incorporating more socially oriented variables and determinants are needed to confirm and expand upon our findings. Additionally, it is crucial to develop and implement targeted interventions and care pathways to enhance health outcomes for frail inpatients with neurological syndromes.

**Table 1:** Deficits included in the adopted 40-item frailty index

40 - Item Frailty Index		
Deficits	Presence = 1	Absence = 0
**Comorbidities**		
1. Hypertension		
2. Arrythmias		
3. Ischemic heart disease		
4. Heart failure		
5. Diabetes mellitus		
6. Chronic kidney disease		
7. Previous stroke		
8. Cognitive impairment/Dementia		
9. Psychiatric disease		
10. Malignancy		
11. Autoimmune disorders		
12. Chronic obstructive pulmonary disease		
13. Endocrinopathies		
14. Chronic infections		
15. Obesity or malnutrition		
**Clinical signs and symptoms**		
16. Abnormalities in cardiac examination		
17. Abnormalities in chest examination		
18. Abnormalities in abdominal examination		
19. Abnormalities in cutaneous examination		
20. Focal neurological signs		
21. Disorders of consciousness		
22. Agitation		
23. Headache		
24. Systolic blood pressure < 90 mmHg or > 140mmHg		
25. Diastolic blood pressure < 50 mmHg or > 90mmHg		
26. Heart rate < 50 bpm or > 100 bpm		
27. Respiratory rate > 20 bpm		
28. Peripheral oxygen saturation < 90%		
29. Temperature > 37.5 °C		
**Laboratory abnormalities**		
30. Hemoglobin < 12 g/dL or > 17 g/dL		
31. Platelets < 50 × 103 or > 450 × 10		
32. White cell count < 4 × 10^3^ or > 10 × 10^3^		
33. Spontaneous INR < 0.8 or > 1.2		
34. ALT < 12 UI/L or > 41 UI/L		
35. Troponins > 0.014 µg/L		
36. CRP > 0.5 mg/dL		
37. Glycemia < 74 mg/dL or > 200 mg/dL		
38. Creatinine > 1.2 mg/dL		
39. Sodium < 136 mEq/L or > 145 mEq/L		
40. Potassium < 3.5 mEq/L or > 5.1 mEq/L		
Total Number Of Deficits		
Frailty Index Score (= n deficits/40, range 0–1)		

**Table 2:** Characteristics and hospitalization outcomes of study participants

**Characteristics**	
Total sample, n	185
Sex (Female), n (%)	93 (50.3)
Age, Mean (SD)	68.6 (18.6)
Frailty Index	
Median [IQR]	0.15 [0.10]
Range	0.00–0.43
Frailty (FI ≥ 0.25), n (%)	31 (16.8)
Diagnosis at hospital discharge, n (%)	
Ischemic stroke	71 (38.4)
Epilepsy	33 (17.8)
Traumatic brain injury	19 (10.3)
Cognitive decline and delirium	17 (9.2)
Hemorrhagic stroke	10 (5.4)
Meningitis and/or Encephalitis	6 (3.2)
Peripheral nerve disease	6 (3.2)
Demyelinating disease	5 (2.7)
Peripheral vertigo	4 (2.2)
Brain neoplasm	3 (1.6)
Cephalalgia	3 (1.6)
Neuromuscular disorder	3 (1.6)
Syncope	2 (1.1)
Other	3 (1.6)
**Hospitalization Outcomes**	
Length of stay (days), Median [IQR]	8.0 [6.5]
Discharge destination, n (%)	
Home	139 (75.1)
Rehabilitation	28 (15.1)
Long-term care	9 (4.9)
Hospice	7 (3.8)
Death, n (%)	2 (1.1)
Nosocomial infections, n (%)	61 (33.0)
Prescription of antibiotics, n (%)	64 (34.6)
Delirium, n (%)	23 (12.4)
Prescription of sedatives, n (%)	27 (14.6)
Prescription of physical restraints, n (%)	34 (18.4)
Falls, n (%)	4 (2.2)

## Abbreviations

CFS: Clinical Frailty Scale
ED: Emergency Department
FI: Frailty Index
